# The effect of localization and histological verification of endometrial polyps on infertility

**DOI:** 10.1007/s00404-019-05155-3

**Published:** 2019-04-11

**Authors:** Judit Lőrincz, Szabolcs Molnár, Attila Jakab, Tünde Herman, Singh Jashanjeet, Péter Török

**Affiliations:** 1 Department of Obstetrics and Gynaecology, Kenézy Gyula University Hospital, Debrecen, Hungary; 2 Faculty of Medicine, Institute of Obstetrics and Gynaecology, University of Debrecen, Debrecen, Hungary; 3 Centre for Assisted Reproduction, University of Debrecen, Debrecen, Hungary

**Keywords:** Endometrial polyps, Infertility, Hysteroscopy, Reproduction

## Abstract

**Aim:**

Our purpose is to investigate if transcervical resection of endometrial polyps improves the fertility in ovulatory infertile women, and whether polyp histology, intrauterine location, and the technique of polypectomy have any influence on the pregnancy rates.

**Methods:**

In this retrospective study, clinical data of 87 ovulatory infertile women who underwent hysteroscopy and polypectomy, and their 12-month follow-up have been analyzed. Subgroups according to the method of polyp removal (resectoscope or curettage), the polyp localization (utero-tubal, anterior, posterior, lateral, multiple) and the histological result were interpreted.

**Results:**

Mean age of patients was 33.99 ± 4.24 years. There were no differences in the BMI and basal FSH levels between the subgroups. Pregnancy was recorded in 30 (34.5%) within the next 12 months without any difference between the subgroups of polypectomy method applied. Posterior wall polyp resection increased the pregnancy chance (OR 5.02), but no other differences were observed in 1-year pregnancy rates to other localizations. Removal of polyps which had normal endometrial histology had lower pregnancy rates as compared to that of polyps with hyperplasia or endometrial polyp histology results (OR 0.25).

**Conclusions:**

Polypectomy improved the conception rate in the subsequent year regardless of the intrauterine localization and the method of its surgical removal. Therefore, we can conclude that polypectomy should be considered in infertile women.

## Introduction

Infertility is “a disease of the reproductive system defined by the failure to achieve a clinical pregnancy after 12 months or more of regular unprotected sexual intercourse” (WHO). Forty percent of the cases of infertility are due to male factors, another 40% are due to female factors, and the remaining 20% are idiopathic. Endometrial polyps are thought to cause uterine factor infertility by disrupting the uterine lining or they may inhibit sperm movement or prevent successful embryo implantation within the uterus [1].

Endometrial polyps are common lesions both in reproductive and postmenopausal ages; they are tissue growths that occur in the uterine cavity, which mainly represents focal hyperplasia of the basal layer of endometrium [2]. Histologically, it is composed of endometrial glands and stroma around a vascular axis of spiral arteries [3]. The pathogenesis of the polyps is not exactly identified; however, it is thought to be similar to endometrial hyperplasia. Hormonal disorders such as anovulation, luteal phase defect, oestrogen excess or oestrogen therapy can affect the formation of endometrial polyps [4, 5]. Polyps may be single or multiple, of various sizes, sessile or pedunculated [2]. Even though most polyps are asymptomatic, they can cause clinical complaints, among which abnormal uterine bleeding is the most frequently reported [6]. Endometrial polyps can be detected by ultrasonography, hysterosonography, hysterosalpingography, endometrial biopsy and uterine curettage, but diagnostic hysteroscopy is considered to be the gold standard method with the greatest sensitivity of 95.3% and specificity of 95.4% [6–10]. Recently, the incidence of endometrial polyps seems to be increasing, due to the increase in its detection rate through imaging techniques applied in the everyday clinical practice [6]. Even though polyps are considered benign lesions, there is no consensus in its management [11]. Some researchers suggest complete removal in all cases; others propose more conservative treatments [12, 13], whereby recommending polypectomy only in symptomatic cases, or in women of postmenopausal age [2, 8].

Asymptomatic endometrial polyps are often found in women being examined for infertility, but their exact role as an etiologic factor is not clear yet. Even though there is poor evidence of the real effect of polypectomy on infertility and live birth rate [14], several studies suggest that the presence of the endometrial polyps can reduce the optimal conditions for implantation [15]. Mittal and co-authors reported that the glands and stroma in endometrial polyps are unresponsive to progesterone stimulation, which can lead to defective implantation at the site of the polyp [16]. The presence of an endometrial polyp may also induce local inflammatory changes or distort the uterine cavity, thus interfering with normal implantation and embryonic development [17]. According to Yanaihara [18], polyps, depending on their tubo-cornual localization can block migration of sperms, causing infertility by ruining the patency of the genital tract. These theories reflect the uncertainty, whether and to what extent polyps influence the fertility through implantation failure.

In this study, we investigated if transcervical resection of endometrial polyp (TCRP) improves the fertility status, and whether polyp histology, intrauterine location, and the technique of polypectomy have any influence on the pregnancy rates (PR) in ovulatory infertile women.

## Materials and methods

A retrospective study of 87 ovulatory infertile women referred for endometrial polyps to our tertiary referral centre along with our county-teaching hospital was conducted between 2014 and 2016. In all of them, female infertility was the indication for detailed clinical infertility work up, after exclusion of the male factor by andrological examination and spermatogram. Upon confirmation of a focal endometrial lesion using either a color Doppler or saline-enhanced ultrasound examination, hysteroscopy was indicated. Only patients with visually confirmed (e.g. hysteroscopically) polypoid structure were included in our study.

The hysteroscopic operations were recorded in the documentation by detailed description, pertaining to the polyp’s localization, its form, size and surface. Moreover, a schematic picture showing the uterus in two dimensions (horizontal and coronal plan) was applied, enabling the operating physician to draw the polyp. In all the cases, after detecting the polypoid structure(s) with hysteroscope, it was removed either by resectoscope, or by curettage according to the choice of the operator. According to literature data, every polyp should be removed regardless of its size [19]. Localization of the polyp in the endometrial cavity was categorized into five groups: utero-tubal junction, anterior uterine wall, posterior uterine wall, lateral uterine wall, and multiple. Multiple polyps were defined as the presence of three or more polyps in more than two locations. All operations were performed under general anaesthesia. Informed consent was signed. During operation resectoscope (Storz, Germany) with a 4 mm 30° optic and 11.5-mm-diameter sheath was used. The electrosurgical system had a 5-mm-diameter 90° electrode. Monopolar technique was used with the output of 60–100 W. For the distension, 1.5% glycine was used with an inflow pressure of 80–100 mmHg. Without preoperative medical preparation, Hegar dilators were used for cervical dilatation up to 11.5 mm. All samples underwent histological examination. Preceding the hysteroscopy, other possible causes of infertility such as anovulation, tubal factor and male factor had been excluded during the infertility work up. All patients were followed, and pregnancies confirmed by ultrasound in the subsequent 1 year after the polypectomy were recorded. The pregnancy rate was compared in the five groups of polyps, as well as according to the resection type and histopathology. Comparative statistics including Student’s *t *test or Mann–Whitney test, and Chi-squared or Fisher's exact tests were used for continuous or categorical variables, respectively. All statistical analyses were performed using SPSS Version 21.0 (IBM Corp, Armonk, NY). Significance was defined as *p* < 0.05.

## Results

The patients’ mean age was 33.99 ± 4.24 years. In 70 cases (80.5%), polyps were removed using a resectoscope, whereas in 17 cases (19.5%) curettage was performed. The average BMI in the resectoscope group was 25.2 and 27.9 in the curettage group. Respectively, the average FSH level in the hysteroscopy group was 7.52 ± 3.93 IU/L and 7.29 ± 1.27 IU/L in the curettage group. In the resectoscope group, histopathology confirmed endometrial polyp in 72.8% (*N* = 51), endometrial hyperplasia in 4.3% (*N* = 3) and in 22.9 (*N* = 16), endometrial polyp or endometrial hyperplasia was not diagnosed. In the curettage group, results were similar: endometrial polyp in 70.6% (*N* = 12), endometrial hyperplasia in 5.9% (*N* = 1) and negative in 23.5% (*N* = 4). Localization of the polyps were as follows: utero-tubal junction 17.2% (*N* = 15); anterior uterine wall 24.2% (*N* = 21); posterior uterine wall 27.6% (*N* = 24); lateral uterine wall 24.2% (*N* = 21); and multiple 6.8% (*N* = 6).

Successful conception was recorded in 30 women (34.5%) in the subsequent 1 year after the intervention. During the follow-up, out of the hysteroscopy group, 23 patients got pregnant in the first year (2 IVF, 21 spontaneous), and 7 women out of the curettage group (7 spontaneous). Patients were divided into subgroups by age, localization of polyps, the removing method and histological results. The 1-year pregnancy rate, odds ratios and differences (*p *value) were calculated in each subgroup (Table 1). There was no significant difference in the pregnancy rate between the different age subgroups. The pregnancy rate in the groups according to the localization of the polyps was 54.17% after posterior wall polyp, 46.67% after utero-tubal polyps, 33.33% after multiple polyps, 19.05% after both anterior wall and lateral wall polyps The anterior wall was selected as a reference, the odds ratio to posterior wall with the highest pregnancy rate was 5.02 (1.29–19.43 CI 95%); this difference was significant (*p* = 0.01). Other localizations did not show significantly higher chance for conception within 1 year. The use of resectoscopic technique was not associated with higher pregnancy rate compared to curettage, as the pregnancy rate was 32.86% and 41.18%, respectively (OR 1.43; 0.48–4.24; *p* = 0.51) (Table 1).

**Table 1:** Pregnancy rates in terms of age, localization of the polyp, histology and removal method

Characteristics	Pregnancy rate	OR (95% CI)	*P *value
Overall	30/87 (34.48%)		
Age at procedure			
30 >>	6/15 (40.00%)	Ref	
30–35	11/32 (34.38%)	0.78 (0.22–2.78)	0.70
35 <<	13/40 (32.50%)	0.72 (0.21–2.46)	0.60
Localization			
Anterior wall	4/21 (19.05%)	Ref	
Posterior wall	13/24 (54.17%)	5.02 (1.29–19.43)	**0.01**
Lateral wall	4/21 (19.05%)	1.00 (0.21–4.66)	1.00
Utero-tubal junction	7/15 (46.67%)	3.71 (0.83–16.47)	0.08
Multiplex localisation	2/6 (33.33%)	2.12 (0.28–15.96)	0.14
Removing method			
Resectoscope	23/70 (32.86%)	Ref	
Curettage	7/17 (41.18%)	1.43 (0.48–4.24)	0.51
Histology			
Simple endometrial polyp	26/63 (41.27%)	Ref	
Endometrial hyperplasia	1/4 (25.00%)	0.47 (0.04–4.81)	0.52
Normal endometrium	3/20 (15.00%)	0.25 (0.06–0.94)	**0.04**

In the pregnancy group of women who underwent polypectomy within a year, histopathology identified endometrial polyp in 26 cases (86.67%), endometrial hyperplasia in 1 case (3.33%) and normal endometrial tissue in 3 cases (10%). Regarding the pregnancy rate in 1 year after the polypectomy, it was higher if histology described simple endometrial polyp (41.27%) than after removing hyperplastic or normal endometrial tissue (25% and 15%). The difference in these results was significant in terms of the extreme values. The removal of polyps histologically with normal endometrium was associated with lower pregnancy rate than removal of classical polyps (OR 0.25; 0.06–0.94 CI 95%; *p* = 0.04) (Table 1).

## Discussion

Endometrial polyps are mostly benign and frequently asymptomatic tumours that are thought to have the potential to interfere with female fertility [20]. Although there is no robust evidence on their relation to female infertility, removal of endometrial polyps in subfertile women is being performed in many countries with the aim to improve the reproductive outcomes. In this study, we found that polypectomy improved the reproductive outcome in the subsequent year, especially if intra-polyp endometrial hyperplasia or endometrial polyp was confirmed by histology. In general, the localization of the polyp might have a smaller effect on the implantation failure; however, after removing the most common posterior wall polyps, the pregnancy rates improved better than in cases where polyps were in the anterior or lateral wall polyps. Although the much less visually controlled curettage was performed in smaller number, the superiority of the resectoscope in this study cannot be confirmed, since the method of polypectomy did not influence the fertility outcomes.

In the last decades due to the improvement and frequent use of the colour or contrast-enhanced ultrasound techniques, the incidence of diagnosis of endometrial polyps has been increasing, as well as their prevalence in the fertility practice. However, hysteroscopy has remained the gold standard of diagnosis with its high sensitivity and specificity as compared to the ultrasonography’s lower specificity [21]. Opinion and attitude of researchers regarding the effectiveness of polypectomy on fertility are far not uniform [15]. In our treatment protocol, infertile, but otherwise asymptomatic ovulatory women are advised to undergo hysteroscopic surgery when intrauterine defect is suspected by imaging. Similarly, to others, in this study we confirmed that reproductive surgery may improve the likelihood of pregnancy, because the uterine receptivity can be increased [13, 16, 22]. Yanaihara et al. reported that location of polyps in uterine cavity is important, after polypectomy the pregnancy rate at the utero-tubal lesions was higher than in case of polyps in other locations [23]. In contrast, we found that the common posterior wall polyps may have larger defective effect on implantation, since pregnancy rate after polypectomy was higher than in other localizations. It has been also reported that even if small polyps (< 2 cm) do not seem to decrease pregnancy rates they may increase pregnancy loss [24]. In the last decades, the development of the hysteroscopic visualization and the electrosurgical instruments for treating uterine conditions made the procedure widely used and safe. Following the see-and-treat principle [25], outpatient polypectomy can be performed without the need for cervical dilation and anaesthesia [26]. Office hysteroscopy has several advantages over traditional methods, such as quick procedure, lower complication of anaesthesia and lower costs [24].

With this study we got an insight into the distribution of polyps according to this localization and histology in infertile women, and the effectiveness of polypectomy. Unfortunately, due to the relatively low number of cases with endometrial polyps, we will continue our data collection prospectively. At this point we can conclude, that regardless of its localization, removal of endometrial polyps diagnosed by hysteroscopy improves the likelihood of successful conception [27]. Based on our data and analysis, histological examination of removed polyps is strongly recommended, as the removal of histologically confirmed macroscopic polypoid structures improved the fertility outcomes.
